# Status of Cardiovascular Health Among Adult Americans in the 50 States and the District of Columbia, 2009

**DOI:** 10.1161/JAHA.112.005371

**Published:** 2012-12-19

**Authors:** Jing Fang, Quanhe Yang, Yuling Hong, Fleetwood Loustalot

**Affiliations:** 1 Division for Heart Disease and Stroke Prevention, National Center for Chronic Disease Prevention and Health Promotion, Centers for Disease Control and Prevention, Atlanta, GA (J.F., Q.Y., Y.H., F.L.)

**Keywords:** cardiovascular health, epidemiology, states

## Abstract

**Background:**

With ideal cardiovascular health metrics, the American Heart Association established a goal of improving cardiovascular health for all Americans by 20% by 2020. Determining how the metrics vary by state is important to the individual states as well as to researchers and policy makers nationwide.

**Methods and Results:**

Using 2009 data from Behavioral Risk Factor Surveillance System, a state‐based telephone survey with 356 441 eligible participants, we examined the 7 metrics defined by the American Heart Association: hypertension, high cholesterol, smoking, body mass index, diabetes, physical activity, and consumption of fruits and vegetables. The 3 primary outcomes of this study were (1) the percentage of the population achieving ideal health status on all 7 factors, (2) the percentage of the population with only 0 to 2 of the 7 metrics (poor cardiovascular health); and (3) the mean overall score (number of ideal metrics). Overall, 3.3% of population was in ideal cardiovascular health, and 9.9% was in poor cardiovascular health. The mean overall score was 4.42. The percentage with ideal cardiovascular health varied from 1.2% (Oklahoma) to 6.9% (District of Columbia ). The adjusted prevalence ratio of ideal cardiovascular health ranged from 0.38, 95% confidence interval 0.29 to 0.52 (Oklahoma), to 1.91, 95% confidence interval 1.51 to 2.42 (District of Columbia), with Illinois as the referent.

**Conclusions:**

In the United States, the cardiovascular health status of the population varies substantially by state. The estimates here could help state programs charged with preventing heart disease and stroke to set their goals for reducing risk and improving cardiovascular health in their jurisdictions.

## Introduction

In the United States, cardiovascular disease (CVD) is the leading cause of mortality, accounting for 1 of every 3 deaths.^1–2^ More than 80 million American adults suffer from this disease.^1^ The economic burden of CVD to our society is significant, with a recent estimate placing the 1‐year cost of this disease, including direct medical costs and indirect costs due to lost productivity from premature death and morbidity at $444 billion.^1,3^ Despite these stark numbers, there has actually been a substantial drop in CVD mortality over the past 4 decades.^2^ Notably, according to a national study,^4^ almost half the reduction in mortality for coronary heart disease, a major subcategory of CVD, from 1980 to 2000 was attributable to a reduction in CVD risk factors (eg, high blood pressure and high cholesterol) and improved health behaviors (eg, not smoking, engaging in regular physical activity, maintaining a healthy diet).^4^ To keep the reduction in mortality sustainable, both primary prevention (treatment and control of risk factors) and primordial prevention (preventing an increase in risk factors in the first place) would be important.

In 2010, the American Heart Association (AHA) adopted the concept of cardiovascular (CV) health in its 2020 Impact Goals.^5^ In the mean time, *Healthy People 2020* developed a new goal: “HDS‐1 (Developmental) Increase overall cardiovascular health in the U.S. population.”^6^ The AHA seeks to improve the CV health of all Americans by a factor of 20% while also reducing deaths from CVD and stroke by 20%.^5^ This is a novel and positive approach, as it encourages the general population to attain outcomes that promote CV health (normal blood pressure, normal cholesterol, no diabetes, and normal weight) while practicing behaviors (no smoking, engaging in adequate physical activity, eating a healthy diet) that have the same goal, and it underscores the importance of preventing the development of risk factors in the first place. The AHA uses 7 metrics to define CV health: smoking status in the past 12 months, body mass index (BMI), levels of physical activity, a measure of dietary intake, and levels of blood pressure, cholesterol, and glucose. Results for each of the 7 metrics are stratified into ideal, intermediate, and poor status.^5^ Published prospective studies in recent years suggest that individuals with 5, 6, or 7 ideal CV health metrics have much lower levels of ischemic heart disease, CV mortality, and all‐cause mortality than do those with 0 to 1 ideal CV health metrics.^7–10^ Currently, however, the prevalence in the US adult population of having 7 ideal CV health metrics is only about 2%, and the level has not changed that much in the past 20 years.^7,9^ To date, differences in the prevalence of having ideal CV health metrics have been examined by age, sex, race/ethnicity, and education,^7,9,11–13^ but to our knowledge, no studies have assessed geographic or regional differences in CV health metrics. The Behavioral Risk Factor Surveillance System (BRFSS) provides the opportunity to measure CV health status at the state level. The objective of the present study was to estimate the state‐specific prevalence of ideal CV health and of poor CV health while also calculating the mean CV health metric score for all 50 states and the District of Columbia using BRFSS data for 2009.

## Methods

### Data

The BRFSS surveys have been conducted annually since 1984 by state departments of health, with assistance from the Centers for Disease Control and Prevention (CDC). BRFSS, the world's largest ongoing telephone health surveillance system, tracks health conditions and health‐related behaviors in all 50 states and the District of Columbia. The survey relies on random‐digit dialing to interview adults aged 18 years or older who are part of the civilian, noninstitutionalized population; detailed information is available at http://www.cdc.gov/brfss. The survey has a core component, optional modules, and state‐added questions. The core component, which is used by all the states, addresses the topics of health‐related perceptions, conditions, and behaviors while obtaining sociodemographic characteristics. Each state decides on its own whether it will use the optional modules, which are developed in coordination with CDC. For the present report, only questions from the core component were used so that the information obtained for every state and DC would cover the same areas. These measures included hypertension, high cholesterol, diabetes, BMI, tobacco use, physical activity, consumption of fruits and vegetables, and cardiovascular prevalence; the 4 demographic characteristics were age, sex, race/ethnicity, and level of education. The median state response rate for the 2009 survey was 52.5% (range 37.9% to 66.9%).

Participants who reported a history of coronary heart disease or stroke were excluded. The 7 CV health metrics are shown in Table 1, which includes the relevant BRFSS questions and the definitions for ideal CV health based on the AHA standards.^5^ Because blood pressure, blood cholesterol, and blood glucose levels could not be directly measured, the indicators for hypertension, high cholesterol, and diabetes were categorized as “no” (ideal) or “yes” based on the self‐reported response. Although the AHA's healthy diet score is based on multiple components—intake of fruits and vegetables, whole grains, sodium, sugar‐sweetened beverages, and fish^5^—this score was not used for the present study. Instead, intake of fruits and vegetables was used; this variable has been employed elsewhere as a proxy for the quality of a diet for cardiovascular health.^14^ The demographic variables included age (18 to 34, 35 to 54, 55 to 64, and ≥65 years), sex, race/ethnicity (non‐Hispanic white, non‐Hispanic black, non‐Hispanic Asian/Native Hawaiian or other Pacific Islander, and non‐Hispanic American Indian or Alaska Native, and Hispanic), and education (less than high school graduate, high school graduate, some college or technical school, and college graduate or above).

**Table 1.** BRFSS Questions and Definitions for Ideal Cardiovascular Health Metrics

Measure	BRFSS Question	Definition for Ideal Cardiovascular Health
Hypertension	Have you ever been told by a doctor, nurse, or other health professional that you have high blood pressure?	Answered “no”
High cholesterol	Those who have cholesterol screened—Have you ever been told by a doctor, nurse, or other health professional that your blood cholesterol is high?	Answered “no”
Glucose	Have you ever been told by a doctor that you have diabetes?	Answered “no”
Body mass index (BMI)	About how much do you weigh without shoes? About how tall are you without shoes?	BMI (kg/m^2^)=18.5 to 24.9
Smoking status	Have you smoked at least 100 cigarettes in your entire life? Do you now smoke cigarettes every day, some days, or not at all? During the past 12 months, have you stopped smoking for 1 day or longer because you were trying to quit smoking? How long has it been since you last smoked cigarettes regularly?	Had not smoked at least 100 cigarettes in their lifetime; or reported smoking 100 cigarettes in their lifetime but not currently smoking.
Physical activity	Now, thinking about the moderate activities you do in a usual week, do you do moderate activities for at least 10 minutes at a time, such as brisk walking, bicycling, vacuuming, gardening, or anything else that causes some increase in breathing or heart rate? How many days/week do you do these moderate activities for at least 10 minutes at a time? On days when you do moderate activities for at least 10 minutes at a time, how much total time per day do you spend doing these activities? Now, thinking about the vigorous activities you do in a usual week, do you do vigorous activities for at least 10 minutes at a time, such as running, aerobics, heavy yard work, or anything else that causes large increases in breathing or heart rate? How many days/week do you do these vigorous activities for at least 10 minutes at a time? On days when you do vigorous activities for at least 10 minutes at a time, how much total time per day do you spend doing these activities?	Did enough moderate or vigorous physical activity to meet the recommendation of ≥150 minutes a week of moderate‐intensity activity, ≥75 minutes of vigorous‐intensity activity, or an equivalent combination of aerobic physical activity.
Healthy diet	Not counting juice, how often do you eat fruit? How often do you eat green salad? How often do you eat potatoes, not including French fries, fried potatoes, or potato chips? How often do you eat carrots? Not counting carrots, potatoes, or salad, how many servings of vegetables do you usually eat?	Consumed 5 or more servings of fruits and vegetables per day

BRFSS indicates Behavioral Risk Factor Surveillance System.

### Statistical Analysis

**Figure 1.**
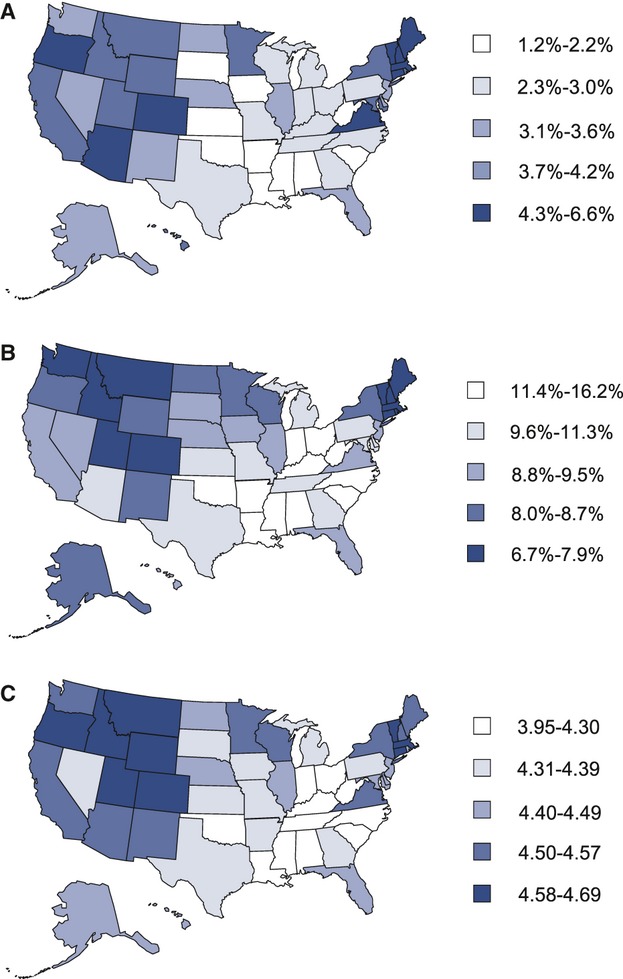
Age‐standardized cardiovascular health status by US states, BRFSS, 2009. A, Age‐standardized prevalence of population with ideal cardiovascular health by states. B, Age‐standardized percentage of population with 0 to 2 cardiovascular health metrics by states. C, Age‐standardized mean score of cardiovascular health metrics by states. BRFSS indicates Behavioral Risk Factor Surveillance System.

**Figure 2.**
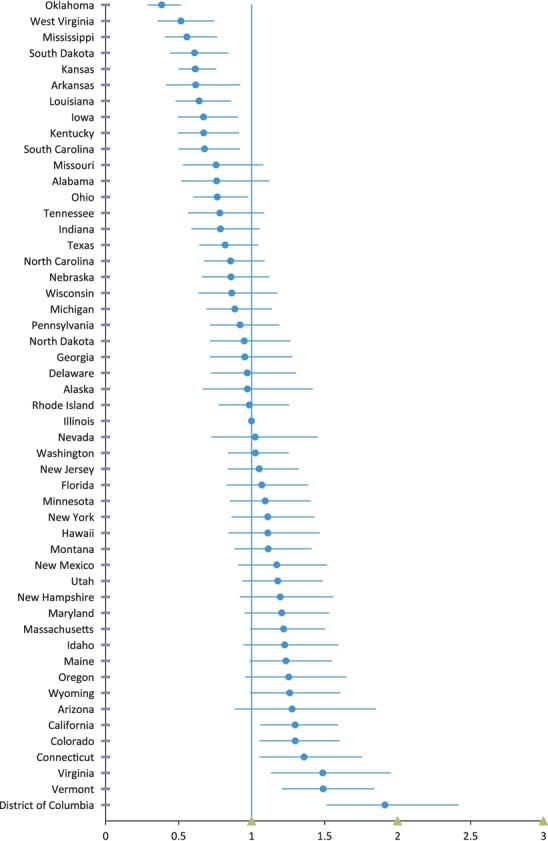
Adjusted prevalence ratio of ideal cardiovascular health by state, BRFSS, 2009. BRFSS indicates Behavioral Risk Factor Surveillance System.

Three measures of CV health are reported in this study: (1) percentage of the population with ideal CV health (designation as “ideal” on all 7 metrics); (2) the CV metrics score, defined as the mean number of “ideal” metrics (possible score of 0 to 7); and (3) poor CV health, defined as the percentage of the population with a score of 0 to 2 ideal metrics. Table 2 presents age‐specific estimates of these values for the 4 age groups as well as age‐standardized estimates (using the 2000 US standard projected population with age distribution 18 to 24, 25 to 44, 45 to 64, and ≥65 years)^15^ for groups categorized by sex, race/ethnicity, and education. Table 3 presents age‐standardized estimates for the 50 states plus the District of Columbia. Figure 1 depicts by state the age‐standardized percentages of the population with ideal CV health and poor CV health and the age‐standardized mean score by state. Figure 2 shows the state‐based adjusted prevalence ratio of ideal CV health, which was determined with a logistic regression model.^16^ In this model, we used Illinois, the state with the median age‐standardized prevalence of ideal CV health, as the referent and adjusted for age, sex, race/ethnicity, and education. All analyses were conducted in a way that accounted for the complex survey design of the BRFSS using SAS‐callable SUDAAN. All reported estimates and 95% confidence intervals (CIs) were weighted using the BRFSS sampling design variables.

**Table 2.** Age‐Standardized* Results for Cardiovascular Health Metrics by Sociodemographic Characteristics, BRFSS 2009

Characteristic	Number	Ideal Cardiovascular Health (All 7 Metrics)	Cardiovascular Health Score (Mean)	Poor Cardiovascular Health (0 to 2 Metrics)
		% (95% CI)	Mean (95% CI)	% (95% CI)
Total	356 441	3.3 (3.2 to 3.4)	4.42 (4.41 to 4.43)	9.9 (9.7 to 10.1)
Age, y				
18 to 34	46 539	3.3 (3.1 to 3.6)	4.88 (4.85 to 4.90)	3.5 (3.2 to 4.0)
35 to 54	132 410	3.8 (3.6 to 4.0)	4.40 (4.38 to 4.41)	9.4 (9.0 to 9.7)
55 to 64	79 527	3.4 (3.2 to 3.5)	3.94 (3.92 to 3.96)	17.4 (16.9 to 17.9)
≥65	97 965	2.2 (2.0 to 2.3)	3.85 (3.84 to 3.87)	18.4 (17.9 to 18.9)
Sex				
Men	131 181	1.9 (1.8 to 2.1)	4.28 (4.26 to 4.30)	10.3 (10.0 to 10.6)
Women	225 260	4.6 (4.4 to 4.7)	4.55 (4.54 to 4.56)	9.4 (9.2 to 9.6)
Race/ethnicity				
Non‐Hispanic white	293 298	3.7 (3.6 to 3.9)	4.48 (4.47 to 4.50)	9.2 (9.0 to 9.4)
Non‐Hispanic black	28 179	1.6 (1.3 to 1.9)	4.05 (4.02 to 4.09)	15.1 (14.3 to 15.9)
Hispanic	22 983	2.0 (1.7 to 2.3)	4.25 (4.21 to 4.28)	11.2 (10.4 to 12.0)
NH Asian/Pacific Islander/Native Hawaiians	7048	4.8 (3.9 to 5.9)	4.65 (4.57 to 4.72)	7.7 (6.5 to 9.2)
NH AI/AN	4933	1.5 (0.8 to 2.5)	4.17 (4.07 to 4.26)	12.2 (10.4 to 14.3)
Education				
<High school	28 942	0.9 (0.7 to 1.1)	3.89 (3.84 to 3.93)	17.3 (16.2 to 18.4)
High school	104 488	1.5 (1.3 to 1.7)	4.13 (4.11 to 4.15)	13.1 (12.7 to 13.7)
Some college	96 255	2.6 (2.4 to 2.8)	4.34 (4.32 to 4.36)	10.2 (9.8 to 10.6)
≥College graduate	126 756	5.6 (5.3 to 5.9)	4.72 (4.70 to 4.74)	6.2 (5.9 to 6.5)

CI indicates confidence interval; BRFSS, Behavioral Risk Factor Surveillance System; NH, non‐Hispanic; AI, American Indian; AN, Alaska Native.

* Age‐standardized applied to total, sex, race/ethnicity, and education, using the 2000 US standard projected population, with age groups 18 to 24, 25 to 44, 45 to 64, and ≥65 years.

**Table 3.** Age‐Standardized Results for Cardiovascular Health Metrics by State, BRFSS, 2009

State	Number	Ideal Cardiovascular Health (All 7 Metrics)			Cardiovascular Health Score (Mean)			Poor Cardiovascular Health (0 to 2 Metrics)		
		%	95% CI		Mean	95% CI		%	95% CI	
Alabama	5575	2.2	1.5	3.2	4.2	4.1	4.2	13.9	12.6	15.3
Alaska	2044	3.3	2.4	4.6	4.4	4.3	4.5	8.0	6.4	9.9
Arizona	4503	4.2	3.1	5.8	4.5	4.4	4.6	10.0	8.2	12.1
Arkansas	3251	2.0	1.4	2.9	4.3	4.3	4.4	12.1	10.5	14.0
California	15 112	3.9	3.5	4.3	4.5	4.5	4.6	8.8	8.1	9.5
Colorado	10 452	4.5	4.0	5.1	4.7	4.6	4.7	6.7	5.9	7.6
Connecticut	5527	5.5	4.5	6.6	4.6	4.6	4.7	7.1	6.2	8.1
Delaware	3638	3.4	2.6	4.3	4.5	4.4	4.5	9.8	8.6	11.1
District of Columbia	3301	6.9	5.9	8.1	4.7	4.6	4.7	8.3	7.3	9.5
Florida	9835	3.5	2.8	4.3	4.5	4.4	4.5	9.2	8.3	10.3
Georgia	4938	2.9	2.2	3.7	4.4	4.3	4.5	10.5	9.4	11.7
Hawaii	4616	3.7	3.1	4.5	4.5	4.4	4.5	8.8	7.7	10.1
Idaho	4591	4.0	3.3	4.8	4.6	4.5	4.7	7.8	6.8	9.0
Illinois	5081	3.4	2.8	4.0	4.4	4.3	4.5	9.4	8.3	10.5
Indiana	7771	2.5	2.0	3.2	4.2	4.2	4.3	11.7	10.6	12.9
Iowa	5204	2.2	1.7	2.8	4.4	4.3	4.4	9.4	8.0	10.9
Kansas	16 085	2.2	1.9	2.5	4.3	4.3	4.4	9.6	9.0	10.2
Kentucky	7663	2.3	1.7	3.0	4.2	4.1	4.2	14.0	12.6	15.4
Louisiana	7260	1.9	1.5	2.4	4.2	4.1	4.2	13.2	12.2	14.3
Maine	6903	4.5	3.8	5.2	4.6	4.5	4.6	7.8	7.1	8.6
Maryland	7295	4.0	3.4	4.8	4.5	4.4	4.5	10.7	9.6	12.0
Massachusetts	14 057	4.6	4.1	5.1	4.6	4.6	4.7	7.6	6.9	8.5
Michigan	7540	3.0	2.5	3.6	4.4	4.3	4.4	10.6	9.6	11.6
Minnesota	4954	4.2	3.5	5.0	4.6	4.5	4.6	8.2	7.1	9.5
Mississippi	9119	1.5	1.2	2.0	4.0	4.0	4.1	14.7	13.8	15.8
Missouri	4182	2.5	1.8	3.5	4.3	4.3	4.4	10.7	9.4	12.2
Montana	6491	3.7	3.1	4.4	4.6	4.5	4.7	7.4	6.4	8.5
Nebraska	13 597	3.1	2.5	3.8	4.5	4.4	4.5	9.0	8.0	10.2
Nevada	3162	3.2	2.3	4.3	4.4	4.3	4.5	9.2	7.7	11.0
New Hampshire	5158	4.5	3.7	5.7	4.5	4.5	4.6	7.9	7.1	8.9
New Jersey	10641	3.6	3.1	4.2	4.5	4.4	4.5	8.8	8.0	9.6
New Mexico	7494	3.6	3.0	4.3	4.6	4.5	4.6	8.1	7.1	9.1
New York	5852	3.7	3.1	4.5	4.5	4.4	4.6	8.7	7.8	9.7
North Carolina	11 133	2.7	2.3	3.1	4.3	4.2	4.3	11.4	10.4	12.4
North Dakota	4087	3.3	2.7	4.1	4.5	4.4	4.5	8.3	7.4	9.4
Ohio	8199	2.5	2.1	3.0	4.3	4.2	4.3	11.7	10.5	13.0
Oklahoma	6159	1.2	0.9	1.5	4.2	4.1	4.2	12.5	11.4	13.6
Oregon	3306	4.3	3.5	5.3	4.6	4.5	4.7	8.2	6.9	9.8
Pennsylvania	7697	3.0	2.5	3.6	4.4	4.3	4.4	10.5	9.5	11.6
Rhode Island	5351	3.7	3.1	4.4	4.5	4.4	4.6	7.8	6.9	8.8
South Carolina	7976	2.1	1.6	2.7	4.3	4.2	4.3	11.7	10.4	13.2
South Dakota	5828	2.1	1.6	2.7	4.4	4.3	4.4	9.5	8.1	11.0
Tennessee	4689	2.3	1.6	3.3	4.2	4.1	4.3	11.2	9.9	12.7
Texas	9828	2.4	2.0	2.8	4.3	4.3	4.4	10.4	9.3	11.6
Utah	8919	3.9	3.4	4.5	4.6	4.6	4.7	7.3	6.5	8.1
Vermont	5836	5.5	4.8	6.2	4.6	4.6	4.7	7.2	6.3	8.3
Virginia	4372	5.0	4.1	6.2	4.6	4.5	4.6	8.8	7.8	10.0
Washington	17 186	3.6	3.3	4.0	4.5	4.5	4.6	7.9	7.4	8.4
West Virginia	3917	1.5	1.1	2.1	4.0	3.9	4.0	16.2	14.7	17.9
Wisconsin	3854	2.8	2.1	3.6	4.5	4.4	4.6	8.3	7.1	9.6
Wyoming	5212	4.2	3.6	5.0	4.6	4.5	4.6	8.7	7.6	9.9

CI indicates confidence interval; BRFSS, Behavioral Risk Factor Surveillance System.

## Results

The 2009 BRFSS survey had 424 592 respondents. After excluding those with coronary heart disease or stroke (n=52 800) as well as those with missing information on age, sex, or race/ethnicity (n=15 351), 356 441 adults remained and were included in the present report. By state, the number of participants ranged from 2044 (Alaska) to 17 186 (Washington).

Overall, 3.3% (95% CI 3.2% to 3.4%) of the population of interest had ideal CV health (Table 2), whereas the mean total CV health score was 4.42 (95% CI 4.41 to 4.43). In all, 9.9% (95% CI 9.7% to 10.1%) of the population had poor CV health (0 to 2 “ideal” health metrics).

We found large disparities in CV health by age, sex, race/ethnicity, and level of education (Table 2). All 3 indicators were significantly different by age, sex, race/ethnicity, and level of education. By age, those who were 65 or older had the lowest percentage of ideal CV health, the lowest CV health score, and the highest percentage of poor CV health. In contrast, the 18‐ to 34‐year age group had both the highest mean score and the lowest percentage of poor CV health, whereas the 35‐ to 54‐year age group had the highest percentage of ideal CV health. In all comparisons, women fared better than men, whereas non‐Hispanic whites and non‐Hispanic Asians/Pacific Islanders consistently performed better than the other 3 race/ethnicity groups. In contrast, on each of the 3 measures, non‐Hispanic blacks and non‐Hispanic American Indians and Alaska Natives fared more poorly than the other 3 groups. In terms of educational attainment, persons with the highest level consistently scored better than the other 3 groups.

By state, the percentage of the population with 7 ideal CV health metrics (a perfect score) ranged from 1.2% in Oklahoma to 6.9% in the District of Columbia (Table 3). The mean CV health score ranged from a low of 4.0 in Mississippi and West Virginia to 4.7 in Colorado and the District of Columbia, and the percentage with poor CV health ranged from 6.7% (Colorado) to 16.2% (West Virginia). In general, Western and New England states had a higher percentage of having all 7 CV metrics be ideal (“ideal CV health”), a higher mean score, and a lower percentage of poor CV health than did the Southern states (Figure 1). Figure 2 reveals that the adjusted prevalence ratio of ideal CV health, using Illinois as the reference, after adjusting for age, sex, race/ethnicity, and level of education ranged from a low of 0.38 in Oklahoma to a high of 1.91 in the District of Columbia.

The age‐standardized percentage for each of the 7 individual CV health metrics is presented in Table 4.

**Table 4.** Age‐Standardized Percentage of Individual Cardiovascular Health Metrics by State, BRFSS, 2009

States	Normal Weight (BMI 18.5–24.9)			Meet 2008 Physical Activity Guideline			Eat ≥5 Fruits or Vegetables/Day			Do Not Smoke			No History of Diabetes			No History of Hypertension			No History of High Cholesterol		
	%	95% CI		%	95% CI		%	95% CI		%	95% CI		%	95% CI		%	95% CI		%	95% CI	
Alabama	31.5	29.5	33.5	60.9	58.7	63.0	20.9	19.1	22.7	78.1	76.2	79.8	89.5	88.5	90.5	66.6	65.0	68.3	67.7	65.6	69.8
Alaska	35.6	32.6	38.8	73.4	70.6	76.1	22.3	19.6	25.1	80.7	78.1	83.0	93.0	91.3	94.5	71.6	69.0	74.0	68.7	65.5	71.8
Arizona	34.6	31.7	37.7	69.6	66.9	72.3	24.4	22.1	27.0	84.6	82.4	86.6	91.4	90.1	92.6	75.1	73.1	77.1	64.9	61.4	68.3
Arkansas	33.2	30.7	35.8	66.2	63.8	68.6	20.0	18.0	22.2	78.9	76.5	81.1	91.0	89.8	92.1	69.7	67.7	71.6	70.2	67.9	72.4
California	37.2	36.2	38.3	67.3	66.2	68.4	27.7	26.7	28.7	87.6	86.8	88.3	90.4	89.8	91.0	75.1	74.2	75.9	67.9	66.7	69.0
Colorado	43.2	41.8	44.6	73.9	72.6	75.1	24.9	23.7	26.2	83.3	82.2	84.4	93.6	93.0	94.2	76.9	75.9	77.8	68.7	67.1	70.2
Connecticut	41.1	39.1	43.2	71.0	69.3	72.7	28.6	26.7	30.5	84.2	82.5	85.9	93.2	92.5	93.9	74.9	73.5	76.4	67.2	65.1	69.3
Delaware	35.4	33.1	37.7	69.8	67.5	72.0	25.3	23.2	27.5	81.6	79.6	83.5	92.5	91.5	93.5	71.5	69.7	73.2	67.5	65.3	69.6
District of Columbia	46.7	44.3	49.0	71.1	69.0	73.0	31.1	29.1	33.3	84.8	83.0	86.5	91.6	90.6	92.6	73.2	71.5	74.8	67.9	65.8	69.8
Florida	37.4	35.4	39.5	68.0	66.1	69.9	24.9	23.1	26.8	81.2	79.3	82.9	91.7	90.7	92.5	73.6	72.1	75.0	68.1	66.2	69.9
Georgia	34.1	31.9	36.3	64.6	62.4	66.8	24.8	22.8	26.9	82.6	80.7	84.3	90.8	89.9	91.7	70.0	68.3	71.6	67.1	64.8	69.3
Hawaii	39.9	38.0	41.7	72.7	71.0	74.3	23.4	21.9	25.0	84.5	83.1	85.8	91.3	90.4	92.1	70.2	68.8	71.6	66.3	64.2	68.4
Idaho	38.6	36.7	40.6	72.8	70.9	74.6	25.0	23.3	26.8	84.3	82.6	85.9	92.5	91.7	93.2	76.1	74.7	77.4	70.1	68.2	71.9
Illinois	35.2	33.4	36.9	66.5	64.7	68.3	22.9	21.4	24.5	81.7	80.1	83.1	92.5	91.6	93.2	71.9	70.4	73.3	67.4	65.4	69.3
Indiana	33.6	32.1	35.2	65.0	63.4	66.5	20.8	19.4	22.2	77.2	75.8	78.6	91.3	90.6	91.9	70.3	69.1	71.5	66.6	65.0	68.1
Iowa	31.7	30.0	33.5	66.9	65.2	68.4	18.3	17.0	19.8	82.8	81.3	84.3	93.2	92.5	93.9	73.9	72.5	75.3	68.6	66.7	70.4
Kansas	34.2	33.2	35.3	65.3	64.3	66.2	18.8	18.1	19.6	82.7	81.8	83.5	92.4	92.0	92.9	72.5	71.7	73.2	67.1	66.0	68.2
Kentucky	33.4	31.4	35.4	65.3	63.4	67.1	21.7	19.9	23.6	75.1	73.2	77.0	90.0	88.9	90.9	66.3	64.7	67.9	65.3	63.3	67.3
Louisiana	31.7	30.1	33.3	62.1	60.5	63.7	17.0	15.7	18.3	78.5	77.0	79.9	89.7	88.8	90.5	65.5	64.1	66.8	69.0	67.4	70.5
Maine	35.7	34.0	37.3	73.2	71.7	74.7	27.8	26.4	29.3	82.3	80.9	83.6	92.8	92.2	93.4	73.2	72.0	74.4	68.5	67.1	69.9
Maryland	36.2	34.5	37.9	66.0	64.3	67.6	27.9	26.3	29.5	85.3	84.0	86.5	90.7	89.7	91.5	71.5	70.1	72.8	66.0	64.2	67.8
Massachusetts	41.8	40.3	43.2	70.7	69.3	72.0	26.4	25.1	27.6	85.2	84.1	86.2	93.0	92.4	93.6	76.2	75.2	77.1	68.7	67.2	70.2
Michigan	33.7	32.3	35.3	68.5	67.0	69.9	22.8	21.5	24.1	80.7	79.4	82.0	91.5	90.8	92.1	72.3	71.2	73.5	67.4	66.0	68.8
Minnesota	36.3	34.4	38.2	68.7	66.9	70.4	22.1	20.5	23.7	83.4	81.7	84.9	93.7	92.9	94.4	77.2	75.8	78.5	70.3	67.9	72.6
Mississippi	28.8	27.4	30.3	57.8	56.2	59.3	16.9	15.7	18.2	77.0	75.5	78.3	89.6	88.9	90.3	64.8	63.5	66.0	66.4	64.9	67.9
Missouri	34.0	31.8	36.2	66.7	64.5	68.8	19.7	17.9	21.6	77.5	75.3	79.5	92.6	91.7	93.4	69.7	67.9	71.4	69.3	67.2	71.3
Montana	37.4	35.5	39.3	74.3	72.8	75.8	25.6	24.0	27.3	83.1	81.5	84.5	94.0	93.3	94.6	74.2	72.8	75.6	70.5	68.5	72.5
Nebraska	35.1	33.3	37.0	67.5	65.8	69.2	20.7	19.4	22.2	83.4	81.9	84.9	92.5	91.8	93.1	74.6	73.4	75.8	69.0	67.3	70.6
Nevada	35.5	32.6	38.5	70.1	67.5	72.7	24.3	21.6	27.3	78.4	75.6	80.9	92.9	91.6	94.1	74.8	72.7	76.8	68.0	64.9	70.9
New Hampshire	37.7	35.6	39.9	70.4	68.5	72.3	28.1	26.1	30.1	84.2	82.3	85.8	93.6	92.8	94.3	73.6	72.0	75.1	66.4	64.2	68.5
New Jersey	37.2	35.7	38.8	65.2	63.7	66.6	26.5	25.2	27.8	84.4	83.2	85.6	91.9	91.1	92.6	73.9	72.7	75.0	67.3	65.8	68.8
New Mexico	36.6	35.0	38.4	70.8	69.2	72.3	23.2	21.9	24.7	82.6	81.2	83.9	91.6	90.8	92.3	74.6	73.3	75.8	71.7	70.2	73.2
New York	39.0	37.2	40.8	65.9	64.1	67.7	27.2	25.6	28.8	82.1	80.6	83.5	91.7	90.9	92.5	73.0	71.5	74.4	66.2	64.3	68.0
North Carolina	33.8	32.3	35.4	65.5	64.0	67.0	21.2	20.0	22.5	80.2	78.9	81.5	91.1	90.3	91.8	69.5	68.3	70.7	66.0	64.4	67.6
North Dakota	31.6	29.7	33.5	67.3	65.4	69.1	22.6	21.0	24.2	81.6	79.9	83.2	93.1	92.2	93.8	75.3	73.9	76.7	71.3	69.4	73.0
Ohio	33.3	31.6	35.0	66.9	65.2	68.5	21.0	19.7	22.3	80.1	78.7	81.5	91.1	90.4	91.8	71.0	69.6	72.2	66.0	64.1	67.8
Oklahoma	32.1	30.5	33.8	64.8	63.2	66.3	14.5	13.4	15.8	75.0	73.5	76.6	90.3	89.5	91.1	68.8	67.5	70.1	67.5	65.8	69.0
Oregon	38.7	36.5	41.0	73.0	70.9	75.0	26.5	24.5	28.6	82.7	80.7	84.6	92.4	91.3	93.3	75.1	73.4	76.7	70.8	68.4	73.1
Pennsylvania	35.2	33.6	36.8	67.0	65.4	68.5	24.1	22.7	25.5	79.5	78.1	80.9	92.1	91.4	92.8	71.0	69.7	72.3	67.4	65.7	69.1
Rhode Island	37.2	35.3	39.1	66.6	64.8	68.4	26.4	24.8	28.0	84.6	83.1	86.0	94.0	93.4	94.6	71.8	70.3	73.2	68.4	66.6	70.1
South Carolina	33.5	31.6	35.4	64.3	62.4	66.1	17.3	16.0	18.7	80.2	78.6	81.8	90.6	89.7	91.4	70.0	68.4	71.5	64.8	62.6	66.9
South Dakota	32.0	30.1	34.0	62.8	60.9	64.7	15.3	14.1	16.7	82.4	80.8	84.0	93.3	92.6	93.9	72.5	71.0	73.9	70.3	68.2	72.3
Tennessee	30.7	28.5	33.0	50.5	48.0	53.1	23.3	21.3	25.4	77.9	75.6	80.1	89.2	88.1	90.3	69.5	67.6	71.4	72.0	69.6	74.2
Texas	32.8	31.0	34.5	65.2	63.4	67.0	23.9	22.5	25.4	82.8	81.3	84.2	90.4	89.5	91.3	70.7	69.4	72.0	63.9	61.8	66.0
Utah	38.8	37.5	40.2	72.8	71.6	74.0	23.8	22.6	25.0	90.7	89.8	91.5	92.3	91.6	92.9	74.2	73.2	75.3	69.6	68.2	70.9
Vermont	41.7	40.0	43.4	74.2	72.6	75.7	29.5	27.9	31.1	82.2	80.7	83.6	94.5	93.9	95.0	74.1	72.9	75.4	70.3	68.4	72.1
Virginia	39.0	36.6	41.4	68.6	66.4	70.8	27.5	25.4	29.7	81.3	79.0	83.3	92.3	91.4	93.1	73.7	71.9	75.3	67.7	65.2	70.0
Washington	37.0	35.9	38.1	70.4	69.4	71.4	25.2	24.3	26.2	85.4	84.6	86.2	92.6	92.1	93.0	72.9	72.1	73.7	68.0	67.0	69.1
West Virginia	31.4	29.5	33.3	49.4	47.4	51.4	16.5	15.1	18.0	73.4	71.5	75.2	89.1	88.0	90.1	66.2	64.5	67.8	67.8	65.8	69.7
Wisconsin	33.6	31.1	36.2	69.5	67.0	71.9	22.2	20.2	24.3	81.0	78.7	83.0	93.0	92.0	93.9	74.3	72.6	76.0	69.9	67.5	72.1
Wyoming	37.0	35.2	38.9	72.9	71.2	74.5	23.6	22.1	25.3	80.5	78.8	82.0	93.7	93.0	94.3	75.5	74.3	76.7	70.0	68.3	71.7

BRFSS indicates Behavioral Risk Factor Surveillance System; BMI, body mass index.

## Discussion

Cardiovascular health is a positive health metric, including factors and behaviors that prevent or protect individuals from heart disease and stroke. Attaining ideal CV health, the optimal health of the heart and vascular system, is a valuable goal for all Americans. The AHA quantified the attainment of this goal through a system of metrics that accord with current health recommendations and guidelines. This report indicates that the percentage of Americans with ideal CV health is low and that there are substantial disparities in CV health metrics among the US population by age, sex, race/ethnicity, and education as well as by state.

Although in this study, having ideal CV health was uncommon, the estimates were, in fact, higher than those from previous reports. For example, data from the Atherosclerosis Risk in Communities (ARIC) study (1987–1989) found that only about 0.1% of participants had ideal CV health.^7^ Another recent report, this one using data from the National Health and Nutrition Examination Survey (NHANES), found that the percentage of the population with ideal CV health was 2.0%, 1.3%, and 1.2% in 1988–1994, 1999–2004, and 2005–2010, respectively.^9^ Two reasons may explain the possible overestimate of ideal health in the BRFSS: (1) BRFSS data are self‐reported, and (2) there were limited data to assess a healthy diet. For example, the 3 health outcome statuses (hypertension, high cholesterol, and diabetes) in the BRFSS were based on self‐reports (yes versus no), whereas these estimates in ARIC and NHANES were based on direct laboratory or blood pressure measurements. In addition, calculations of the BMI in the BRFSS from self‐reports could result in underestimates of overweight or obesity. As evidence, in NHANES (which uses technicians to measure height and weight) for 2001–2006, participants in general tended to overreport their actual height (as determined by measurement), whereas men tended to overreport their actual height and women tended to overreport their height and underreport their weight.^17^

**Figure 3.**
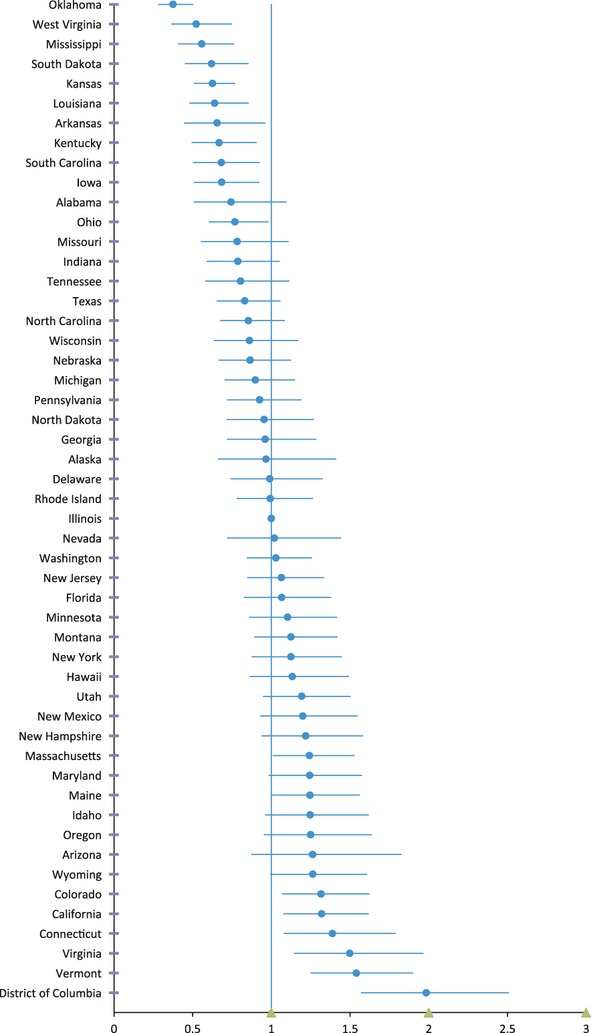
Adjusted prevalence ratio of ideal cardiovascular health by state, including patients with coronary heart disease and stroke, BRFSS, 2009. BRFSS indicates Behavioral Risk Factor Surveillance System.

In this report, we excluding those with self‐reported coronary heart disease and stroke, as cardiovascular risk status among patients with coronary heart disease and stroke differ from those of general population. This would overestimate ideal CV health in the overall population. We conducted separate analyses including those with self‐reported coronary heart disease and stroke. Although the percentage of ideal CV health was lower than among those excluding those with prevalent disease, the disparities by age, sex, and race/ethnicity were the same (Table 5). For states, some changed position from the median state (Figure 3).

**Table 5.** Age‐Standardized* Results for Cardiovascular Health Metrics by Sociodemographic Characteristics, Including Participants With History of Coronary Heart Disease and Stroke, BRFSS, 2009

	Number	Ideal Cardiovascular Health (All 7 Metrics)		Cardiovascular Health Score (Mean)		Poor Cardiovascular Health (0 to 2 Metrics)	
		% (95% CI)	*P* Value	Mean (95% CI)	*P* Value	% (95% CI)	*P* Value
Total	40 6498	3.1 (3.0 to 3.2)		4.34 (4.33 to 4.35)		11.4 (11.2 to 11.6)	
Age, y							
18 to 34*	47 274	3.3 (3.0 to 3.6)		4.87 (4.84 to 4.89)		3.6 (3.2 to 4.1)	
35 to 54	139 361	3.6 (3.5 to 3.8)	0.034	4.33 (4.32 to 4.35)	<0.001	10.7 (10.4 to 11.1)	<0.001
55 to 64	90 537	2.6 (2.4 to 2.7)	<0.001	3.79 (3.78 to 3.81)	<0.001	20.9 (20.4 to 21.4)	<0.001
≥65	129 326	1.7 (1.6 to 1.9)	<0.001	3.68 (3.67 to 3.71)	<0.001	21.9 (21.4 to 22.3)	<0.001
Sex							
Men*	154 224	1.8 (1.6 to 1.9)		4.20 (4.18 to 4.22)		12.1 (11.8 to 12.4)	
Women	252 274	4.4 (4.2 to 4.5)	<0.001	4.48 (4.47 to 4.50)	<0.001	10.8 (10.5 to 11.0)	<0.001
Race/ethnicity							
Non‐Hispanic white*	335 392	3.6 (3.4 to 3.7)		4.41 (4.40 to 4.42)		10.7 (10.5 to 10.9)	
Non‐Hispanic black	32 455	1.5 (1.3 to 1.8)	<0.001	3.98 (3.93 to 4.01)	<0.001	17.2 (16.4 to 18.0)	<0.001
Hispanics	25 224	1.9 (1.6 to 2.1)	<0.001	4.17 (4.14 to 4.21)	<0.001	12.8 (12.0 to 13.6)	<0.001
NH Asian/Pacific Islander	7560	4.6 (3.8 to 5.6)	0.03	4.59 (4.51 to 4.66)	<0.001	8.6 (7.4 to 10.0)	0.003
NH AI/AN	5867	1.3 (0.7 to 2.3)	<0.001	4.04 (3.95 to 4.13)	<0.001	14.9 (13.2 to 16.9)	<0.001
Education							
<High school*	37 305	0.8 (0.6 to 1.0)		3.77 (3.73 to 3.82)		20.4 (19.3 to 21.4)	
High school	122 357	1.4 (1.2 to 1.6)	<0.001	4.05 (4.03 to 4.08)	<0.001	14.9 (14.5 to 15.4)	<0.001
Some college	109 116	2.4 (2.2 to 2.6)	<0.001	4.26 (4.24 to 4.28)	<0.001	11.8 (11.5 to 12.2)	<0.001
≥College	137 720	5.4 (5.1 to 5.7)	<0.001	4.67 (4.65 to 4.69)	<0.001	7.1 (6.8 to 7.3)	<0.001

CI indicates confidence interval; NH, non‐Hispanic; BRFSS, Behavioral Risk Factor Surveillance System; AI, American Indian; AN, Alaska Native.

* Age‐standardization applied to total, sex, race/ethnicity, and education using the 2000 US standard projected population, with age groups 18 to 24, 25 to 44, 45 to 64, and ≥65 years.

* *P* values for differences across the categories and for comparisons with the reference group.

In fact, potential underestimates of health status, including hypertension, high cholesterol, and obesity, are unavoidable in surveys that depend on self‐reports because of participants' lack of awareness.^18^ In the present study, presumed underestimates of these conditions would most likely have resulted in overestimates of ideal CV health. In addition, we did not use the metric for an ideal healthy diet employed by the AHA, which was based on the adoption of 4 or 5 of 5 components of a healthy diet: fruit and vegetable intake ≥5 servings per day; two 3.5‐oz servings of fish per week; ≥3 servings of 1 oz of whole grains per day; <1500 mg per day of sodium intake, and ≤36 ounces of sugar‐sweetened beverages per week.^5^ Instead, the present report used intake of fruits and vegetables as a proxy for a healthy diet, as it was available from the BRFSS. In a report from the ARIC study that used data from that cohort, only 5.3% met the ideal healthy diet as defined by the AHA,^7^ whereas we estimated from the BRFSS that 24% of the population of interest met the modified definition of an ideal healthy diet used in this survey (consumption of 5 or more servings of fruits or vegetables per day).^19^

Although the differences in the definitions no doubt affected the estimated prevalence of the CV health metrics, the reported disparities were, in general, consistent with prior reports.^20–22^ Non‐Hispanic whites and non‐Hispanic Asians or Pacific Islanders/Native Hawaiians and those with a higher level of education had better CV health than non‐Hispanic blacks and non‐Hispanic American Indians/Alaska Natives and those with a lower level of education.^20,23^ Adults from the Southern states were less likely to be in ideal CV health, a finding consistent with studies reporting a higher prevalence of chronic diseases and risk factors in Southern states (eg, tobacco use,^24^ obesity,^25^ hypertension, and diabetes^26^). Similar to the findings from studies conducted in the 1990s that examined the correlation between the prevalence of multiple CVD risk factors and coronary heart disease mortality among US states,^27–28^ we found that the prevalence of CV health metrics was significantly correlated with all‐cause, CVD, and cerebrovascular disease mortality using 2008 data from the National Vital Statistics System,^2^ with *r*=−0.712, −0.639, and −0.677, respectively (*P*<0.001), among the 50 states.

In our report, the highest percentage among the states or the District of Columbia of ideal CV health was found in the District of Columbia, and 2 individual metrics (Table 4) for the District of Columiba give further credence to that ranking. The District of Columbia had the highest percentage of the population with normal weight and eating ≥5 fruits and vegetables/day, which are the 2 metrics with the lowest percentage in generally. In fact, recent reports showed that the District of Columbia was 1 of the 2 states with an obesity rate lower than 20%^25^ and had the highest percentage of intake of 2 or more servings of fruits and the second highest percentage of intake of 3 or more servings of vegetables.^29^

As a population‐based, cross‐sectional survey performed across the United States each year, the BRFSS offers the strengths of a large sample and the ability to examine data for each state. Perhaps its most important limitation in terms of producing accurate estimates is its reliance on self‐reports. In the present study, this reliance on self‐reporting suggests 2 cautions: first, the need to be mindful of the general principle that self‐reporting contains bias; and second, the problem that certain categories (hypertension, high cholesterol, diabetes, and BMI) that should be measured objectively^5^ were instead calibrated simply by the participants' responses. In effect, this kept this investigation from optimally assessing the full range of the AHA categories of “ideal CV health,” “intermediate CV health,” and “poor CV health.” In addition, the 2009 survey that was used for the present report included only residents with a landline telephone and excluded those in institutions and long‐term care facilities. Finally, the 52.5% median response rate in the present study, although acceptable, could have limited the generalizability of the results if nonrespondents systematically differed from respondents in their answers. Regardless, with the state‐based sample survey, the BRFSS provides the opportunity to report state‐level health indicators, which are usually not available in other national surveys.

In conclusion, this report provides estimates of CV health for all 50 US states and the District of Columbia. The data can be used by state programs as a baseline assessment of CV health. The use of BRFSS data to assess CV health highlights the flexibility of the BRFSS to assess new and emerging health classifications and to document disparities by sociodemographic status and geographic region. Although the BRFSS has methodological limitations as it relates to the traditional AHA definition of CV health, the findings of low levels of ideal CV health and the patterns of disparities are consistent with the findings of other researchers. Numerous national‐, state‐, and community‐level activities support the pursuit of CV health. For example, the CDC State Heart Disease and Stroke Prevention Programs currently fund 41 states and the District of Columbia to build state capacity, enhance surveillance and monitoring, and facilitate population‐level activities that improve CV health. In addition, numerous population‐level programs support the promotion of CV health by developing and implementing evidence‐based strategies across a wide variety of populations, including the Racial and Ethnic Approaches to Community Health program, the Community Transformation Grants program,^30^ and the Communities Putting Prevention to Work^31^ program. Findings from this report can be used by stakeholders to direct communication initiatives, focus limited resources, and support programmatic plans to improve CV health.
